# Study on the Impact of Environmental Tax on Industrial Green Transformation

**DOI:** 10.3390/ijerph192416749

**Published:** 2022-12-13

**Authors:** Yang Shen, Xiuwu Zhang

**Affiliations:** Institute of Quantitative Economics, Huaqiao University, Xiamen 361021, China

**Keywords:** resource tax, environmental regulation, green development, punishment for trust-breaking, industrial agglomeration

## Abstract

Tax revenue is one of the essential means through which the government controls the macro-economy and plays a vital role in promoting environmental protection and sustainable development. This study takes Chinese panel data from 2004 to 2020 as sample observations, uses the SBM-GML index method to measure industrial green total factor productivity, and then uses econometric methods such as the two-way fixed effects model and instrumental variable method to analyze the impact of an environmental tax on industrial green transformation. It is found that the generalized environmental tax represented by vehicle and vessel tax, resource tax, and urban land use tax has a significant positive effect on industrial green transformation. After a series of robustness tests and the exclusion of endogeneity, this conclusion remains valid. The research shows that credit governance, the agglomeration of producer service, and their co-agglomeration with manufacturing are important adjustment mechanisms. Among them, credit management is special and compulsory, greatly restricting the environmental pollution behavior of industrial enterprises, and encourages enterprises to make green investments and to actively improve production processes.

## 1. Introduction

Since humanity entered the era of industrialization, the ecological environment has been not only the most oversized public good, but has also become the most easily damaged good [1,2]. Unlike the industrialization process in developed countries, China has developed from an agricultural country to a country in the middle and late stages of industrialization stage, taking only a few decades to complete the industrialization process that took developed economies hundreds of years. China is a latecomer to the worldwide industrial revolution and represents a miracle of rapid industrial development and long-term social stability. The modernization of China’s manufacturing industry is a strategic model of time for space, which determines that the industrial development model must be a “high-input, high-consumption, high-emission” extensive economy. With the rapid development of industry, China’s manufacturing industry is facing huge problems, such as resource shortages, air pollution, and the destruction of the ecological environment, which seriously restrict sustainable economic development. Green development is the key to solving the bottleneck of resources, energy, and environment and is the only way to promote the intensive development of the manufacturing industry. Currently, China is transitioning from the middle stage of industrialization to the late. Accelerating green industrial development has become the only way to promote ecological progress, a new type of industrialization, and high-quality development [3,4]. The Made in China 2025 initiative, launched in 2015, has fully implemented the relevant requirements for ecological progress. Putting forward the “comprehensive implementation of green manufacturing” as a critical task called for strengthening the promotion and application of energy-saving and environmental protection technologies, processes, and equipment. We will further promote cleaner production and implement the “green manufacturing project”. How to coordinate industrial adjustment, pollution control, and ecological protection; promote carbon reduction, pollution reduction, green expansion, and growth; and promote ecological priority, conservation, and intensive green development have practical significance.

Fiscal and taxation policies are essential to promoting green development and helping to reduce pollution and carbon emissions [5,6]. The negative externalities of the environment mean that green development is unable to rely on a single market regulation mechanism. The environmental regulation tool is an effective external driving force. It can exert cost pressure on enterprises, thus making industrial enterprises produce green products. The introduction of environment-related taxes plays a guiding function through the multiplier effect of “directional induction” and “rent creation”. It has an impact on the industrial structure of the manufacturing industry. Taking the resource tax as the representative, through the ad valorem levy, a direct adjustment mechanism linking the resource tax with the resource price is established, which will guide market players to comprehensively develop and utilize resources and improve total factor productivity. It is the primary source of industrial energy consumption and environmental pollutants. In a sense, promoting the green development of industry is the only way that the grand blueprint for building a beautiful China to be realized smoothly. Since the development of the industrial sector has aggravated resource consumption and environmental pollution, this fact is clear. Thus, starting from the industrial sector, exploring the impact of ET on the green production of industrial enterprises will help to accelerate the high-quality development of China’s manufacturing industry.

## 2. Literature Review

ET originated from the theories of negative externalities proposed by welfare economists. After the upsurge of tax reform in western countries, it was widely introduced into tax systems in many countries in the late 1990s [7]. Because of the purpose of green tax, it has a natural connection with economic growth and green development. Some scholars have discussed the economic effect of green tax. 

According to the “compliance cost” proposed by Gray and Shadbegian [8], some scholars believe that the collection of environmental tax will increase the cost of enterprises, distort the allocation of resources, and hinder the improvement of green total factor productivity [9]. With the wide application of the CGE model in the environmental tax effect, this viewpoint has been further strengthened. Bovenberg and Mooij [10] believed that in an economic environment with tax distortions, the introduction of environmental taxes aggravated factor distortions, which strengthened the marginal social damage caused by environmental pollution and was not conducive to the improvement of economic performance and environmental performance. Yuan and Xiang [11] investigated the impact of environmental regulation on green development by constructing an extended Crepon–Duguet–Mairesse (CDM) model and found that environmental regulation promoted the improvement of energy efficiency and labor productivity in the short term. However, this model only improves energy efficiency and hampers labor productivity in the long run. Chintrakarn [12] posits that the cost pressure caused by environmental regulation was the main reason for the low technical efficiency of the manufacturing industry. Richardson and Chanwai believe that the industrial energy tax implemented in the Northwest of England has not led to significant investment in energy efficiency technologies or in renewable fuels and that the tax has had a relatively modest and discretionary social impact [13]. 

Other scholars, based on the “innovation compensation” proposed by Porter [14], believe that collecting various environmental taxes helps improve environmental quality and production efficiency. That is, there is a “double dividend” of environmental taxes. Green fiscal and taxation policies can effectively stimulate the rapid development of products such as clean energy, low-carbon industry, green technology, and natural carbon sinks. Jaffe and Palmer [15] proposed the “narrow Porter hypothesis” based on Porter’s theory, that is, that flexible regulation produces a greater “incentive effect” for innovation. Li and Shi [16] proposed an improved Super-SBM model with unintended output and found that government regulation significantly improved energy efficiency through model calculation, which verified the correctness of the Porter hypothesis to a certain extent. Karydas and Zhang [17] found that green taxation can stimulate innovation and promote economic growth in the long run, even with diminishing margins. Wang et al. found that a carbon tax could reduce greenhouse gas emissions by 8.6% in the short term and reduce PM2.5 emissions by 0.9% and 5.7% in the short and long term, respectively [18]. Fan et al. [19] believe that when implementing environmental tax policy, firms will be motivated to invest more into pollution control because the cost of penalties on firms rapidly increases as the environmental tax rate rises, the negative impact of pollution on business production continues to deepen, and the marginal return on energy input begins to fall. 

In conclusion, there are many studies in the existing literature that focus on the impact of an environmental tax on environmental quality and reach certain accepted conclusions. However, the impact of an environmental tax on economic growth still needs to be further deepened, especially with regard to the lack of direct evidence of industrial development. Therefore, this study verifies the impact of an environmental tax on green industrial transformation from the perspective of environmental regulation. From the perspective of innovation, credit governance and industrial agglomeration are introduced as moderating variables and a statistical model is used to seriously assess the multiple impact mechanism of an environmental tax on green industrial production. Finally, in the robustness test, a policy evaluation of the water resource taxes levied in 2016 and 2017 is conducted.

## 3. Theoretical Framework and Research Hypotheses

### 3.1. The Direct Impact of ET on IGT

Pollution has a negative externality, so it is difficult to solve its adverse effects by relying solely on market forces [20]. The primary purpose of the environmental tax is to encourage companies to change their production strategies, use more environmentally friendly technologies, encourage consumers to use clean energy, and reduce emissions, thus reducing the harm to the environment [21,22]. Different from the viewpoint of neo-classical economics that rising costs crowd out profits, Porter’s hypothesis examines the innovation compensation effect of environmental regulation on improving business performance from the dynamic perspective of the enterprise life cycle [23]. According to this theory, well-designed environmental regulation tools can encourage enterprises to carry out green R&D activities and enhance their market competitiveness, thus partially or even wholly offsetting the cost increase or profit reduction brought about by environmental regulations. As the hypothesis of “Double Dividend” assumes, environmental tax improves environmental quality (the green dividend) and reduces tax distortions (the blue dividend) at the same time. Revenues from environmental taxes are recycled to correct other distortions in the economy, which also create more jobs and reduce economic inefficiencies [24,25,26]. The New Keynesians further extended the Porter hypothesis by pointing out that environmental regulation can help restrain the “self-control” problem caused by the current interest preferences of enterprise agents and stimulate their innovative investment.

Theoretically speaking, with the help of administrative power, we can achieve the goal of preventing environmental pollution and realizing green transformation. However, the environmental regulation tool of administrative order cannot promote sustainable energy conservation and emission reduction. When emissions meet government limits, companies lose the incentive to buy clean equipment or innovate green technology. In addition, administrative control is mainly driven by the government, and the government and the public usually bear its cost, so the implementation cost is high. Environmental tax implements the “polluter pays principle”, the “paid use principle” and other related requirements, and the net environmental policy is organically integrated with economic policy. The ecological environmental service fees, environmental damage fees, and pollution control fees directly affect the commodity price, service prices, and various prices. Environmental tax, in the form of price guidance, transmits technological innovation and cleaner production as favorable market signals to enterprises. Suppose the tax that enterprises pay due to pollution is much higher than the cost of pollution control. In that case, enterprises will be more inclined towards technological innovation and the use of environmental protection equipment. At the same time, environmental tax can also realize recycling by subsidizing renewable energy projects and energy-saving technologies to promote the development of renewable technologies [27]. Therefore, environmental tax increases the costs of an enterprise through economic incentives, and its influence on the manufacturing industry is mainly manifested in the form of “Porter effect” enterprises with high pollution, high energy consumption, and low efficiency, which are forced to withdraw from the market or make green transformations because of the cost constraints of an environmental tax. Environmental constraints have raised the threshold of market entry, making it difficult for potentially inefficient and energy-intensive enterprises to enter the market. The production and operation of clean enterprises are less affected by environmental constraints, and the regional average green total factor productivity is therefore improved. According to the pollution refugee hypothesis, introducing an environmental tax will cause the high-polluting enterprises in the region to transform into enterprises with relatively loose environmental supervision. It will also reduce the number of polluting enterprises within the jurisdiction in order to achieve the purpose of environmental protection. Based on the above discussion, hypothesis 1 is proposed.

**Hypothesis** **1:** *Environmental taxes can promote the green transformation of industry.*

### 3.2. The Adjustment Effect of Credit Management

The institution-based view creates a dynamic interaction between institutions and organizations, and managers and enterprises make rational decisions under institutional constraints [28]. In order to survive and progress in a competitive market, enterprises need to achieve their set goals and be bound by a formal system. With regard to the pure public goods of the ecological environment, it is even more necessary for administrative departments to formulate and perfect related environmental supervision policies and strengthen law enforcement. Regulatory measures such as tax incentives, income and pollution charges, and environmental disclosure cannot ultimately promote China’s industrial green transformation, although these measures have achieved good results in other countries [29,30]. There are many reasons for this predicament. From the government’s point of view, since the reform of the commercial system, the number of market players has increased dramatically, while the regulatory authorities are limited by the number of law enforcement officers and the lack of detection technology, as well as the fact that it is difficult to identify potential illegal pollution. From the enterprises’ point of view, green innovation requires sustained and stable financial support, but endogenous financing is generally challenging when it comes to meeting innovation needs. Technological transformation under the constraints of capital will occupy the capital elements in other production links. As long as the output income is higher than the emission cost, rational decision-makers will not take the initiative to make green investments. Unlike market-motivated environmental regulation tools, green credit and credit control policies are highly specific and mandatory, which profoundly impact industrial enterprises’ environmental pollution behavior. 

From 2013, China’s environmental authorities, as well as financial and other departments, will jointly punish economic entities that have been found, based on information obtained, to have behaved in a way that threatened in environmental protection. Once on the trust-breaking governance list, the production and operation of enterprises will be seriously affected. Not only will they suffer from frequent supervisory spot checks, but they will also be “vetoed by one vote” when dealing with public affairs, and they may also face stricter financing constraints [31]. Yin et al. [32] believe that collective punishment has dramatically increased the cost of environmental violation and dishonesty. For enterprises caught breaking the promise by a correctional center, their marginal cost will increase sharply, and their social image will be affected. In contrast, loyal subjects will be able to enjoy green channels or preferential resources in market transactions, government procurement, and capital acquisition. The environmental credit system not only evaluates the environmental behavior of enterprises, but also the environmental ethics, environmental attitude, and environmental behavior preparation of enterprises [33]. After implementing the green credit and trust-breaking punishment policies, some heavily polluting enterprises will actively fulfill their social responsibilities and communicate their “green” signal to the public through a good environmental, social, and governance (ESG) performance. Flammer [34] believes that green bonds can be used as a reliable signal of enterprises to the environment. Issuing green bonds is not a superficial “floating green” tool, but enhances the green R&D investments of enterprises. Generally speaking, credit-based supervision, rewards, and punishment have an obvious target orientation, which makes enterprises more willing to eliminate outdated equipment and actively improve production technology. Credit governance is a helpful supplement to the non-mandatory features of green taxation and helps stimulate the endogenous power of the green transformation of industrial enterprises. Based on the above discussion, hypothesis 2 is proposed.

**Hypothesis** **2:** *Credit management can strengthen the positive relationship between green taxation and green transformation of industry*.

### 3.3. Regulating Role of Industrial Agglomeration

The theory of the new industrial zone points out that the enterprise organizations gathered in the production complex of a social region can form a highly specialized division of labor or subcontract to form a long-term stable relationship based on mutual trust. This division of labor and cooperation helps to spread knowledge and information among enterprises and brings about the efficiency and mobility that a single producer cannot realize, thus extending the industrial value chain. From the perspective of resources, industrial agglomeration and its scale effects are important driving forces for regional economic growth. The rapid development of the regional economy promotes the centralized utilization of production factors such as the labor force, capital, technology, and data. Enterprises in the agglomeration area gradually become symbionts. Firms within the region share infrastructure, capital externalities, and pools of labor reserves [35]. This sharing mode enables related technical information to be quickly and accurately converted into design specifications and equipment parameters, improves the flexibility of both assets and the specialization of production methods, and helps to improve the efficiency of resource utilization. Under the influence of technological externalities and joint actions, enterprises in clusters enjoy higher collective efficiency. Tacit knowledge and skilled workers can interact and communicate more freely within the industry, which helps to reduce the average technological innovation cost of a single enterprise and further improve the overall innovation capability in the region. Therefore, industrial agglomeration can affect industrial output efficiency, reduce environmental pollution and energy consumption, and promote green industrial transformation through scale effects and technological progress.

According to the theory of industrial symbiosis and agglomeration, enterprises clustered in a specific range have the advantages of circular integration, green technology externalities, and increasing new returns through energy exchange, material exchange, and information exchange to realize the organic unity of economic effects and environmental protection [36]. Environmental deterioration and resource depletion have seriously weakened the competitive advantages of traditional enterprise clusters. The continuous strengthening of environmental regulations has also increased the cost of enterprises and incurred increased transportation costs. In order to deal with industrial waste, enterprises with symbiotic relationships in the agglomeration area will spontaneously organize themselves to establish cleaner production and metabolic chains. By continuously strengthening the concentration degree of related industries in the cluster area, it can attract a venous industry cluster of waste management and resource reuse and realize symbiotic benefits within the cluster relationship. This will also promote the recycling of resources and materials and the green transformation of industry. As a knowledge-intensive industry, producer services have a certain advantage in that it is easier to form knowledge collisions and knowledge networks, which helps enterprises break through the shackles of “face-to-face communication” in traditional agglomeration theory. The spillover effect of sharing labor force and knowledge information will therefore be more pronounced, which will help enterprises to form a good innovation environment and business model [37]. 

Ellison and Glaeser [38] first defined the spatial agglomeration of heterogeneous related industries as industrial synergy agglomeration. The producer service industry has the inherent characteristics of high industrial agglomeration, strong professionalism, an obvious driving effect and high innovation activity, which helps to improve the overall innovation efficiency of the region, promote the deep integration of the modern service industry and the traditional manufacturing industry, and promote manufacturing enterprises to extend themselves towards both ends of the “smile curve” in the production process, so as to achieve efficient, clean, and intensive industrial development. Traditional high-energy-consuming enterprises are forced to do so by market competition, and the interaction between various service enterprises such as high-pollution control logistics, finance, and management is strong. The agglomeration of the producer services industry can provide a one-stop service for the whole process and the entire industry chain for manufacturing enterprises in order to, to a certain extent, reduce the intermediate investment of the manufacturing industry, improve the resource conversion rate, and realize more efficient collaborative production [39,40]. Based on the above discussion, hypotheses 3 and 4 are proposed.

**Hypothesis** **3:** *The agglomeration of producer services can strengthen the positive relationship between green tax and the green transformation of industry*.

**Hypothesis** **4:** *Collaborative agglomeration of manufacturing and producer services can strengthen the positive relationship between green taxation and industrial green transformation.*

## 4. Research Design

### 4.1. Variable Description

(1) Core explanatory variable. The core explanatory variable is environmental tax (ET). Environmental tax is the total of environmental tax, resource tax, and other taxes related to the environment levied on market entities to promote environmental protection, rationally develop and utilize natural resources, and maintain ecological balance [41]. According to the intensity and purpose of taxing environmental protection, environmental taxes can be divided into broad and narrow statistical dimensions. The general environmental tax policy was not initially set up to protect the environment, but it has an environmental protection function. The narrow environmental tax policy refers to the taxes with obvious pertinence to the environmental function for environmental protection, such as energy tax, transportation tax, and carbon tax. Given the short implementation period of China’s environmental protection tax, the narrow environmental tax cannot meet the requirements of long-term data series analysis. This study, instead, chooses a generalized environmental tax system based on the ideas of Deng [42] and Wang [43]. Generally speaking, the taxes that help prevent pollution and reduce resource waste include consumption tax, resource tax, urban maintenance and construction tax, vehicle purchase tax, vehicle and vessel tax, urban land use tax, cultivated land occupation tax, and sewage charges. Due to the severe lack of data on sewage charges and farmland occupation taxes in the sample statistics period, they were eliminated in this study.
(2) Control variables. Since there are many external factors affecting industrial green transformation [44,45,46,47,48], eight external factors are selected as control variables in this study to minimize the interference of confounding variables on causal effect estimation and obtain more accurate fitting results. Industry density (ID): measured using the ratio of the secondary and tertiary industries’ added value to the urban built-up zone exemption. Economic development level (EDL): measured using real GDP per capita based on 2004 price levels. Environmental governance (EG): measured using the amount of investment completed for industrial pollution control. Energy structure (ES): measured using industrial coal consumption as a proportion of total energy consumption. Macro-control (MC): measured using the proportion of government public general budget expenditure to gross national product. Opening degree (OD): measured by the proportion of the total import and export trade (according to the domestic destination and source of goods) converted from the average exchange rate of RMB to USD over the years to the local gross national product. Road accessibility (RA): measured by road mileage. Population density (PD): measured as the number of people per square kilometer.
(3) Explained variable. The explained variable is industrial green transformation (IGT). The concrete manifestation of the green transformation of industry is that industrial enterprises can produce more financial products by using the same resource elements as before because of the improvement of management efficiency and technological innovation and can, simultaneously, reduce environmental pollution. The idea of investigating the relationship between industrial pollution discharge and economic output is that environmental pollutants are directly regarded as a negative utility output, in which the output of “good” products is maximized, and the output of “bad” products is minimized in the production function. In order to avoid statistical errors caused by traditional measurement methods as much as possible, this study uses an SBM (Slack-Based Model) directional distance function combined with the GML index (Global Malmquist–Luenberger) to measure IGT. This method can deal not only with “good” outputs, but also with the dynamic continuity of “bad” outputs and input elements more scientifically. The measurement method of industrial transformation continues Cobb Douglas’s theory of production function. The average annual labor force, net fixed assets at the end of the year, industrial water consumption, and total energy consumption (a standard ton of coal) of enterprises above the designated size are selected as input variables. The expected output is measured by industrial added value, while the unexpected output is measured by industrial sulfur dioxide and industrial wastewater discharge.
(4) Mediating variable. The mechanism variables mainly include credit governance, professional agglomeration of producer services, and its co-agglomeration with producer services and manufacturing.

Credit management (CM). This is measured by the interest expense of high-energy-consuming industries. Generally speaking, the higher the credit rating of environmental protection, the easier it is for market participants to obtain credit funds to alleviate the dilemma of capital flow shortage. Therefore, green credit has the terminal incentive of credit. Green finance is measured by 1 minus the proportion of interest expenses of six energy-consuming industries. 

The measurement of industrial agglomeration mainly includes primacy index, industrial concentration, spatial Gini coefficient and location entropy. For the purpose of research and considering the availability of data, this study uses the location entropy method to measure the agglomeration degree of producer services. The more employees in a certain industry in a certain area, the denser the distribution of enterprises in that industry in that area. The calculation process expression of industrial agglomeration is $APS=\left(PS_{pt}/PS_{t}\right)/\left(P_{pt}/P_{t}\right)$. APS represents the agglomeration of producer services. $PS_{pt}$ indicates the employment number of producer services in p area in t year. $P_{pt}$ indicates the total number of people employed in p area in t year. $PS_{t}$ indicates the number of employed people in the national producer service industry in t year. $P_{t}$ represents the total number of national employees in t year. The larger the APS, the higher the concentration of producer services.

Collaborative Agglomeration (CA). The agglomeration of industries with relevance and heterogeneity in a certain space has created a situation of collaborative agglomeration. The calculation process of collaborative agglomeration of manufacturing and producer services is $CA=\left[1-\left|APS-MA\right|/APS+MA\right]+\left(APS+MA\right)$. Among them, MA represents manufacturing agglomeration. 

### 4.2. Statistical Model

Firstly, the SBM-GML index model is introduced to measure the green transformation of industry. Consider an economic system with n independent decision-making units (DMUs), each of which has m production factors $x_{q}\left(q=1,2,\cdots ,m\right)$,g kind of expected outputs $y_{r}\left(r=1,2,\cdots ,g\right)$, h kind of unexpected output $b_{f}\left(f=1,2,\cdots ,h\right)$. Then, $x_{qk}$, $y_{rk}$, and $b_{fk}$ represent input i, expected output r, and undesired output f of $DMU_{k}$ respectively. In Euclidean space, the input direction vector and output direction vector are used to set the projection direction $z=\left(z_{x},z_{y},z_{b}\right)$. The form of the distance function is:

(1) $${\vec{D}}_{0}=\left(x,y,b;z_{x},z_{y},-z_{b}\right)=\max\left[\omega \left|\left(y+\omega z_{g},b-\omega z_{b}\right)\in p\left(x\right)\right.\right]$$

In Equation (1), ω represents the value of distance function. Using linear programming to solve the direction distance function of the kth DMU in the period *t*, the following equation can be obtained:

(2) $$\vec{D_{k}^{t}}\left(x_{k}^{t},y_{k}^{t},b_{k}^{t}\right)=\max\omega$$

The constraints are:

(3) $$\left\{\begin{array}{l} \sum_{k=1}^{n}\sum_{t=1}^{T}\lambda_{k}^{t}x_{qk}^{t}+\omega z_{x}\leq x_{k}^{t}\\ \sum_{k=1}^{n}\sum_{t=1}^{T}\lambda_{k}^{t}y_{rk}^{t}-\omega z_{y}\geq y_{k}^{t}\\ \sum_{k=1}^{n}\sum_{t=1}^{T}\lambda_{k}^{t}b_{fk}^{t}-\omega z_{b}\leq b_{k}^{t}\\ \lambda_{k}^{t}\geq 0,k=1,2,\cdots ,n \end{array}\right.$$

$\lambda_{k}^{t}$ stands for weight. Considering the same technology frontier of the global reference, the GML index of period t to *t* + 1 is constructed, and the following can be obtained:

(4) $$GML_{t}^{t+1}={\left[\frac{1+\vec{D}\left(x_{t},y_{t},b_{t};y_{t},-b_{t}\right)}{1+\vec{D}\left(x_{t+1},y_{t+1},b_{t+1};y_{t+1},-b_{t+1}\right)}\times \frac{1+{\vec{D}}_{t+1}\left(x_{t},y_{t},b_{t};y_{t},-b_{t}\right)}{1+{\vec{D}}_{t}\left(x_{t+1},y_{t+1},b_{t+1};y_{t+1},-b_{t+1}\right)}\right]}^{1/2}$$

x, y, and b represent input factors, desired output, and undesired output, respectively.

Then, we introduce the multiple linear models. In order to verify the statistical correlation between environmental tax and a green industrial transition, combined with the theoretical analysis and research hypothesis above, this study constructed the following multiple linear regression model:

(5) $$IGT_{it}=\delta_{0}+\alpha_{1}ET_{it}+\sum_{j=1}^{8}\gamma Control_{ijt}+\mu_{i}+\nu_{t}+\varepsilon_{it}$$

In Equation (5), $\delta_{0}$ is the intercept term, $\alpha_{1}$ is the fitting parameter of environmental tax, *i* and *t* represent individual and time, respectively, $\nu_{t}$ is a time fixed effect, $\mu_{i}$ is individual fixed effects, and $\varepsilon_{it}$ is the random disturbance term subject to the white noise process. *Control* is the information set of control variables, which contains all control variables. j represents the jth control variable, and γ is the estimated coefficient of the control variable. In order to investigate the moderating effects of credit governance and industrial agglomeration on the impact of environmental tax on industrial green transition, this study incorporated mechanism variables into the model in the form of interaction terms based on Equation (5). The following three statistical models were obtained:

(6) $$IGT_{it}=\delta_{0}+\beta_{1}ET_{it}+\beta_{2}ET_{it}\times CM_{it}+\beta_{3}CM_{it}+\sum_{j=1}^{8}\gamma Control_{ijt}+\mu_{i}+\nu_{t}+\varepsilon_{it}$$

(7) $$IGT_{it}=\delta_{0}+\rho_{1}ET_{it}+\rho_{2}ET_{it}\times APS_{it}+\rho_{3}APS_{it}+\sum_{j=1}^{8}\gamma Control_{ijt}+\mu_{i}+\nu_{t}+\varepsilon_{it}$$

(8) $$IGT_{it}=\delta_{0}+\eta_{1}ET_{it}+\eta_{2}ET_{it}\times CA_{it}+\eta_{3}CA_{it}+\sum_{j=1}^{8}\gamma Control_{ijt}+\mu_{i}+\nu_{t}+\varepsilon_{it}$$

In Equations (6)–(8), ET×CM, ET×APS, and ET×CA are the interaction terms between environmental taxes and moderating variables, and the significance and sign direction of their estimated parameters are the foci of this study.

### 4.3. Data Source

Due to the severe lack of data for many variables in the Tibet Autonomous Region of China, we do not consider including Tibet in the statistical sample. At the same time, the statistical caliber of Hong Kong, Macao, and Taiwan is inconsistent with that of the Chinese mainland, and we also removed samples from these three regions. Following the principles of data availability and scientificity, the statistical sample of this study is composed of the panel data of 30 provinces in China from 2004 to 2020. The original data of relevant variables mainly come from the China Statistical Yearbook, China Industrial Statistical Yearbook, China Tax Yearbook, China Environmental Statistical Yearbook, China Economic Census Yearbook (2018), and the EPS (Express Professional Superior) global statistical data platform. Some missing values were filled in by linear interpolation. It should be noted that to reduce the order of magnitude of variables and prevent heteroscedasticity problems, logarithmic processing was carried out for some explanatory variables with large values as part of the process of proper calculation. A descriptive statistical analysis of each variable is provided in Table 1.

## 5. Empirical Analysis

### 5.1. Analysis of the Results of Baseline Regression

The commonly used fitting models of the panel data model include the mixed least squares method, random effects model, and fixed effects model; therefore, the most suitable method for the sample data in this study needed to be tested further. The results of the Hausman and F-test show that the null hypothesis was rejected at the 1% level, and the fixed effects model was considered the most suitable for the sample data in this study. In order to prevent the negative influence of multicollinearity among variables on the fitting results, a multicollinearity test was also carried out. The results show that the maximum variance inflation factor (VIF) is 9.57, the minimum variance inflation factor is 1.28, and the average variance inflation factor is 4.04. These results show that there is no multicollinearity problem in the sample data. 

Table 2 reports the test results of the impact of ET on IGT. Column (1) reports the calculation results only including the variable of ET, but not including control variables, individual fixed effects or time fixed effects. Column (2) reports the calculation results when adding control variables on the basis of column (1). Column (3) reports the settlement results of EI influence on IGT after the individual fixed effects are added. Column (4) adds control variables on the basis of column (3). Column (5) reports the estimated results of an ET on IGT after adding individual fixed and time-fixed effects. Column (6) shows the calculation results with all control variables added. The calculation results in column (6) are discussed as the benchmark regression results, and the ET calculation results reported in this column are the foci of this study. Considering this study’s use of long-term panel data, cross-section correlation, sequence correlation, and heteroscedasticity are inevitable. In this study, the SCC model was used to modify the FE model, as the modified FE model is more suitable for short panel data [49].

It can be seen from Table 2 that the calculation results of all models show that the relevant effects of the ET results on IGT are significantly positive. As such, the more control variables and fixed effects are added to the model, the greater the correlation coefficient between ET and IGT will be. The results of two-way FE show that the correlation coefficient between ET and IGT is 1.238, meaning that it passed the 1% significance test. The results show that ET can internalize the external negative effects of environmental pollution and resource consumption. As a result, hypothesis 1 is strongly confirmed. Collecting ET can reduce pollutant emissions and improve environmental quality by influencing the behavior of producers and consumers. As far as producers are concerned, the introduction of ET has increased the tax burden on industrial enterprises, especially those that generate a large amount of pollution. To eliminate the decrease in profit caused by the increase in tax burden, enterprises should introduce, research and develop environmental protection technologies and gradually replace polluting processes and products with clean processes and green products in order to reduce pollutant emissions. For consumers, it is clear that the introduction of environmental taxes has raised the price of polluting consumer goods. Based on the substitution effect, consumers should reduce their purchases of polluting goods and increase their purchases of environmentally friendly goods. This will increase the derivative demand for the green transformation of industrial enterprises.

### 5.2. Robustness Test

The above results preliminarily verify that ET can promote the IGT, but whether this conclusion is sound remains doubtful. In order to prove the robustness and consistency of the benchmark regression results, the following aspects must be verified.

The first involves the changing of the measurement method of core explanatory variables. The vehicle purchase tax evolved from a fee to a tax based on the vehicle purchase surcharge. This levies taxes on taxable cars and motorcycles purchased by economic individuals. The purpose of levying a travel tax is for the local government to raise funds for renovating public roads and waterways within its jurisdiction. The above two items are included in the broad scope of the ET because although the original purpose of levying travel tax and vehicle purchase tax is not to protect the environment and save resources, they objectively inhibit consumers’ willingness to use motor vehicles and ships. Refined oil is a supplement to vehicles and vessels. Reducing the sales volume of vehicles and vessels leads to the indirect consumption of oil resources. Considering that the categories and outputs of new energy vehicles in the market have gradually increased in recent years, the market share of traditional fuel vehicles is being eroded by new energy vehicles. As the process becomes more mature due to the growth of factories and the pressure of market competition increases, major manufacturers will compete to launch vehicles with higher prices. However, lower purchase prices also decrease the amount of tax paid by consumers. The combined effect of the two forces limits the protective effect of travel tax and vehicle purchase tax on resource consumption. In the new calculation method employed in this study, the income of these two taxes is no longer counted. The calculation method is changed to the proportion of total government revenue that comprises environmental tax in order to reduce the amount of information that is changed between the new variable and the original variable as much as possible.

The second relates to replacing the environmental tax with a more specific ET. In 2006, China issued relevant regulations on the administration of water resource fees, which to a certain extent, improved the efficiency of industrial water use and promoted the conservation, protection, management, and rational development and utilization of water resources. However, the water-saving effect of water resource fees was limited due to the disadvantages of nonstandard collection standards and weak collection rigidity. In order to comprehensively promote the resource tax reform, the State Taxation Administration of The People’s Republic of China announced, in 2016, a pilot project for water resource tax reform in Hebei Province. The water resource tax adopts the method of changing the water resource fee into a tax and brings surface water and groundwater into the scope of taxation. Implementing a quota levy for the high water consumption industry, over-planned water use, and groundwater overdraft area access to groundwater was also deemed appropriate in order to raise the tax standard. In 2017, the water resource tax reform pilot area was expanded to include Beijing, Tianjin, and Inner Mongolia. The water resource tax reform has increased the water costs for industrial enterprises. It can, therefore, force enterprises to change their technological processes and innovations and develop in the direction of circular sustainability. Therefore, this study replaces ET with the water resource tax, taking the pilot areas of water resource reform announced in 2016 and 2017 as the experimental group and the remaining areas as the control group. The staggered double difference method is used for the fitting calculations.

The third involves replacing the statistical model. The first law of geography indicates that all things are related to their neighbors, and the closer things are, the more related they are than things that are farther away. When there is a spatial correlation between economic variables, the traditional econometric methods, such as the mixed least squares method and fixed effects model, cannot obtain unbiased estimations. In order to estimate formula (1) more accurately, this study adopts the spatial Durbin model to modify the traditional fixed effects model. The spatial weight matrix has a great influence on the spatial measurement model. In order to fully reflect the spatial autocorrelation and spatial heterogeneity of IGT, this study selects the economic distance of per capita GDP of each province from 2004 to 2020 and the geographical distance calculated by latitude and longitude among the provincial capitals to form an economic and geographic nested matrix as the weight matrix of the spatial econometric model.

As can be seen from Table 3, the results of the three testing methods all show that ET can positively affect the IGT, which verifies the robustness of the benchmark regression results. Although the significance of the ET estimation coefficient in Method 1 and Method 2 is reduced from 1% of the benchmark regression to 5%, it is still within the acceptable range and does not pose a threat to the robustness of the results. According to the results of the benchmark regression, the fitting coefficient of ET will be smaller in a spatial econometric model with spatial correlation. This confirms the correctness of geo-economic theory and shows a spatial correlation between regional pollution behavior and industrial transformation and upgrading.

### 5.3. Endogenous Problem

This study selects more control variables to reduce the negative impact caused by important missing variables. The two-way fixed effects model allows regional effects to be related to explanatory variables, which can overcome the endogenous problems caused by missing variables to some extent. However, the unbiased estimation result of the model is also threatened by the endogenous causal relationship between the variables. ET is a tax system for environmental pollution and resource consumption that is closely related to the clean technologies used by industrial enterprises. Intuitively, the governments of the big firms that emit large amounts of environmental pollution may adopt stricter environmental control policies, urging firms to make greener changes with higher tax rates. In the case of less industrial pollution and low resource consumption, the regulatory authorities will be more lenient on industrial enterprises, and the corresponding ET rates may be lower for those regions dominated by technology-intensive manufacturing enterprises. In order to strengthen the causal logic of ET to promote IGT, this study chooses the instrumental variable method to verify the results. Concerning the problem of instrument variables (IV), mainly referring to the idea of Batick, a Batick instrument variable is constructed interactively by using the first-order lag term and the first-order difference term of ET [50]. At the same time, to prevent the problem of weak instrumental variables, the second-order lag term of the ET is selected as the second instrumental variable. As can be seen from Table 4, the results of the first stage of the two-stage least square method (2SLS) show that the impact of the two instrument variables on the ET is significantly positive, indicating that the instrument variables we selected meet the relevance principle. At the same time, the F statistic of the weak identification test is 308.14, which is greater than 19.93 of the 10% critical value. It is considered that there is no weak instrument variable problem. The P value of the underidentification test strongly rejects the original assumption that the tool variable is not identifiable. The P value of the sargan test does not reject the original assumption that there is no over-identification. The results of the second stage of 2SLS show that the estimation coefficient of the ET is 1.341, meaning that it passed the 1% significance test. The results show that the promotion effect of an ET on IGT is still valid after overcoming endogenous problems.

Because it is challenging to find a strictly exclusive tool variable in economics, the exogenous nature of the tool variable is always questioned. As such, it is worth relaxing the strict exogenous constraint on the tool variables and regarding them as slightly endogenous variables, and then using the approximate exogenous tool variable method proposed by Conley et al. [51] to re-fit the calculation. Because the model is only allowed to be used in precisely identified cases, and the time lag term is not allowed to be used as the tool variable, only the Batick tool variable is used for calculation here. From the approximate exogenous IV results in Table 4, it can be seen that the estimation coefficient of ET on IGT is 1.530, meaning that it passed the 5% significance test, which proves that the estimation result of 2SLS is reliable.

### 5.4. Test of Influence Mechanism

In order to verify research hypotheses 2 to 4, the econometric models constructed by formulas (6) to (8) are combined, and the results in Table 5 are obtained using the two-way FE model.

As can be seen from Table 5, the estimation coefficient of ET on IGT is always significantly positive. The interaction between CM and ET has an estimation coefficient of 1.392 for IGT, meaning that it passed the 1% significance test. In addition, the estimation coefficient of the interaction between APS and ET is 0.059, meaning that it passed the 5% significance test. Moreover, the estimated interaction coefficient between CA and ET is 0.094, which passed the 10% significance test. The estimation coefficients of the three interactive terms are all positive. Combined with the testing process of regulatory effects, the conclusion can be drawn that the above three channels positively strengthen the positive correlation between ET and IGT, and research hypotheses 2 to 4 are verified. Discipline for breaking one’s promise, credit policy, knowledge spillovers, and scale effects are essential mechanisms to regulate ET and promote the green transformation of industry. By comparing the coefficients of interaction terms, found that the estimation coefficient of interaction terms of CM is significantly more significant than the other two mechanism variables. The results show that credit management measures, such as credit control and environmental blacklisting, have a more significant impact on industrial enterprises and can promote the green transformation of industry.

## 6. Conclusions 

As an essential tool for environmental governance, taxation is essential in promoting ecological civilization and is a green transformation process [52]. In this study, using a two-way fixed effects model, we investigated the multiple impact mechanisms of ET on IGT based on panel data for 30 Chinese provinces from 2004 to 2020. We found that ET in the form of price guidance on green innovation and cleaner production is released to firms as a clear signal that can significantly contribute to the green transformation of industry. Our findings remain after a series of robustness tests and after dealing with endogeneity issues. The results of the mechanism test indicate that CM, APS, and CA are essential mechanisms to enhance ET for the green transformation of industry. According to the empirical results of the mechanism path, CM plays the highest positive regulatory role in environmental tax affecting industrial green transformation. It is very effective to use green financial and credit policies and taxation policies to promote environmental protection. Cutting-edge knowledge and advanced technology are important driving forces for improving production efficiency and green development. APS tends to form specialized intermediate products and service markets, providing specialized cleaner production and pollution reduction solutions for manufacturing enterprises. At the same time, specialization agglomeration reduces transaction costs, which is beneficial to the communication and cooperation within and between productive service enterprises and promotes the deepening of specialization and green transformation of industrial enterprises. CA can enhance economies of scale, reduce energy consumption per unit output through increasing returns to scale, promote the centralized consumption of resources and the centralized treatment of pollutants, build a circular economic system, and improve the efficiency of resource allocation and the green development level of the manufacturing industry. Relevant measures for environmental protection reinforce the innovation compensation effect of green tax and stimulate industrial enterprises to optimize production processes and increase green investments. We found that methods such as green standards, pollutant emission standards, permit systems, sewage charges, and ET can all play a positive role in a system of measures to promote green industrial development. Only the scope of application and the policy effects of different measures differ. An environmental protection tax would not completely solve all the problems involving the green transformation of industry, but specific areas of application and limitations of intervention effects do exist. 

This paper argues that in promoting green industrial development, ET mainly applies to areas that can be regulated by taxation, such as energy consumption, pollutant emissions, and green consumption taxes. By internalizing resources, taxation corrects the external diseconomies of pollutant emission behavior and incentivizes companies to increase green total factor productivity. It positively promotes the application of environmental technology research and development, the promotion of new energy sources, and the development of clean industries with the help of market regulation. 

In order to better encourage environmental taxation to promote IGT, based on the theoretical and empirical results, this paper puts forward the following recommendations: First, improve the clean energy taxation support system. At the legal level, it is recommended to improve the environmental protection tax system based on environmental and energy taxes and to revise and improve the Environmental Protection Tax Law. At the policy level, the scope of environmental protection tax coverage should be further expanded, and the tax rate and levy scope should be adjusted in due course. Second, optimize the industrial layout. According to the main functional area planning requirements, regional resource and environmental carrying capacity, industrial development for classification guidance, clear industrial development direction, development intensity, the construction of outstanding characteristics, dislocation development, and complementary and mutual progress should represent the new patterns of industrial development. Third, strengthen the leading role of credit. The interconnection of information between banks, financial institutions, and environmental departments should be strengthened, and comprehensive research should be conducted, with judgments made on the contributions of enterprises to environmental protection and social responsibility with the help of professional third-party environmental risk evaluation agencies.

This study explores the impact of an environmental tax on industrial green transformation from both theoretical and empirical perspectives, enriching the research on market-based environmental regulatory instruments and externalities and the research framework of its effects on green development. In addition, empirical analyses of a series of econometric models can effectively identify the role of an environmental tax in China’s industrial green development in order to provide a reference for the implementation of China’s industrial green development strategy and the rational development of an environmental tax system. However, there are still some limitations. First, due to the late implementation of the environmental tax policy in China, the original intention of setting the environmental tax variable used in this study is not directly to protect the environment, but to take into account the function of environmental protection in the implementation process. Future research should use specific taxes, such as water resources and environmental taxes, to make policy assessments regarding how they affect the green transformation of industry. Secondly, the policy game between different administrative regions and the degree of economic ties should be considered. Studying the impact of environmental taxes on green industrial development from a spatial perspective may have more policy implications. Third, we used a province-level panel dataset because of the unavailability of city-level data. Finally, it is worth pointing out that this study attempted to provide new evidence on the impact of an environmental tax on green industrial transformation, while measuring IGT represents a challenging issue. Due to data availability, we employed industrial green total factor productivity as a proxy of green industrial transformation, which has some potential limitations. Future research will aim to find ways in which to better measure green industrial transformation. Of course, the limitations do not cast doubt on the results of this study, but they should be addressed in future studies.

## Figures and Tables

**Table 1:** Variable descriptive statistics.

Variable	Variable Symbol	Mean Value	Standard Deviation	Minimum Value	Maximum Value
Industrial green transformation	IGT	1.977	1.223	0.520	7.482
Environmental tax	ET	5.721	1.092	1.665	7.765
Economic development level	EDL	9.613	0.560	8.346	11.313
Population density	PD	7.758	0.576	5.226	8.751
Industry density	ID	1.862	0.778	0.401	3.994
Energy structure	ES	2.524	2.036	0.030	10.626
Opening degree	OD	−1.665	0.967	−4.944	0.509
Macro-control	MC	−1.545	0.419	−2.425	−0.277
Road accessibility	RA	13.239	7.716	0.780	39.440
Environmental governance	EG	−5.749	0.831	−9.368	−3.474
Agglomeration of producer services	APS	1.006	0.333	0.635	2.562
Credit management	CM	0.452	0.142	0.094	0.808
Collaborative agglomeration	CA	2.657	0.453	1.739	3.975

**Table 2:** Results of benchmark regression.

Variable	(1) POLS	(2) POLS	(3) FE	(4) FE	(5) Two-Way FE	(6) Two-Way FE
ET	0.243 *** (5.77)	0.574 *** (5.30)	0.645 *** (8.95)	0.911 *** (5.07)	1.172 *** (5.81)	1.238 *** (5.17)
GDP		−0.017 (−0.10)		7.077 *** (6.75)		6.331 *** (3.23)
PD		−0.132 * (−1.74)		0.299 *** (3.86)		0.387 *** (4.14)
ID		−0.347 ** (−2.09)		−1.464 *** (−6.51)		−1.357 *** (−4.58)
ES		−0.194 *** (−5.95)		−0.253 *** (−5.95)		−0.294 *** (−6.92)
OD		−0.083 (−0.81)		−0.097 (−1.24)		−0.216 ** (−2.60)
MC		0.820 *** (5.24)		−2.991 *** (−6.30)		−2.557 *** (−4.88)
RA		0.031 * (1.91)		0.043 *** (6.16)		0.064 *** (9.74)
EG		0.018 (0.23)		−0.075 * (−1.74)		−0.052 (−1.09)
Individual effects	No	No	Yes	Yes	Yes	Yes
Time effects	No	No	No	No	Yes	Yes
F	25.03 ***	23.97 ***	80.08 ***	91.37 ***	4.24 ***	242.51 ***
R-square	0.0470	0.3014	0.3758	0.5739	0.4363	0.6032
Hausman test			15.57 ***	94.03 ***	21.49 ***	36.65 ***

Note: ***, **, and * are significant at the 1%, 5%, and 10% levels, respectively. The T statistics are reported in parentheses.

**Table 3:** Results of the robustness test.

Variable	(1)	(2)	(3)
ET	0.646 ** (2.68)	0.168 ** (2.29)	1.184 *** (5.98)
Control variables	Yes	Yes	Yes
Individual effect	Yes	Yes	Yes
Time effect	Yes	Yes	Yes
R-square	0.6214	0.6060	0.6109

Note: *** and ** are significant at the 1% and 5% levels, respectively. T statistics are reported in parentheses in columns (1)(2). The z statistics are reported in column (3).

**Table 4:** Endogenous test results.

Variable	2SLS: First Stage	2SLS: Second Stage	Plausibly IV
ET		1.341 *** (4.91)	1.530 ** (2.03)
IV 1	0.151 *** (16.52)		
IV 2	0.641 *** (21.55)	0.089 ** (2.20)	
Weak identification test		308.14	
Underidentification test		255.70 ***	
Sargan statistic		0.3442	
Control variables	Yes	Yes	Yes
Individual effect	Yes	Yes	Yes
Time effect	Yes	Yes	Yes
R-square	0.9877	0.5545	

Note: *** and ** are significant at the 1% and 5% levels, respectively.

**Table 5:** The results of the mechanism test.

Variable	(1)	(2)	(3)
ET	1.298 *** (5.54)	1.266 *** (5.70)	1.232 *** (5.45)
CM	0.479 *** (2.86)		
APS		1.902 ** (2.37)	
CA			0.358 ** (2.11)
ET×CM	1.392 *** (3.93)		
ET×APS		0.059 ** (2.48)	
ET×CA			0.094 * (1.88)
Control variables	Yes	Yes	Yes
Individual effect	Yes	Yes	Yes
Time effect	Yes	Yes	Yes
R-square	0.6224	0.6095	0.6112

Note: ***, **, and * are significant at the 1%, 5%, and 10% levels, respectively. The T statistics are reported in parentheses.

## Data Availability

All of the data are publicly available, and proper sources are cited in the text.
